# Hyaluronic acid-mediated epithelial-mesenchymal transition of human lens epithelial cells via CD44 and TGF-β2

**DOI:** 10.5935/0004-2749.2023-0163

**Published:** 2024-07-09

**Authors:** Yumin Gui, Jianjun Peng, Shuanghong Jiang

**Affiliations:** 1 Wuhan Puren Hospital, Qingshan District Jianshe Fourth Road Benxi Street Te #1, Wuhan, Hubei Province, China; 2 Wuhan University Renmin Hospital, Wuchang District Ziyang Road 22, Wuhan, Hubei Province, China

**Keywords:** Hyaluronic acid, CD44, TGF-β2, Epithelial-mesenchymal transition, Epithelial cells, Capsule opacification

## Abstract

**Purpose:**

The epithelial–mesenchymal transition of human lens epithelial cells plays a role in posterior capsule opacification, a fibrotic process that leads to a common type of cataract. Hyaluronic acid has been implicated in this fibrosis. Studies have investigated the role of transforming growth factor (TGF)-β2 in epithelial–mesenchymal transition. However, the role of TGF-β2 in hyaluronic acid-mediated fibrosis of lens epithelial cell remains unknown. We here examined the role of TGF-β2 in the hyaluronic acid-mediated epithelial–mesenchymal transition of lens epithelial cells.

**Methods:**

Cultured human lens epithelial cells (HLEB3) were infected with CD44-siRNA by using the Lipofectamine 3000 transfection reagent. The CCK-8 kit was used to measure cell viability, and the scratch assay was used to determine cell migration. Cell oxidative stress was analyzed in a dichloro-dihydro-fluorescein diacetate assay and by using a flow cytometer. The TGF-β2 level in HLEB3 cells was examined through immunohistochemical staining. The TGF-β2 protein level was determined through western blotting. mRNA expression levels were determined through quantitative real-time polymerase chain reaction.

**Results:**

Treatment with hyaluronic acid (1.0 µM, 24 h) increased the epithelial–mesenchymal transition of HLEB3 cells. The increase in TGF-β2 levels corresponded to an increase in CD44 levels in the culture medium. However, blocking the CD44 function significantly reduced the TGF-β2-mediated epithelial–mesenchymal transition response of HLEB3 cells.

**Conclusions:**

Our study showed that both CD44 and TGF-β2 are critical contributors to the hyaluronic acid-mediated epithelial-mesenchymal transition of lens epithelial cells, and that TGF-β2 in epithelial-mesenchymal transition is regulated by CD44. These results suggest that CD44 could be used as a target for preventing hyaluronic acid-induced posterior capsule opacification. Our findings suggest that CD44/TGF-β2 is crucial for the hyaluronic acid-induced epithelial-mesenchymal transition of lens epithelial cells.

## INTRODUCTION

Cataracts are the leading cause of vision loss in older populations worldwide. In 2012, the World Health Organization estimated that 28 million people in China are visually impaired due to cataracts^(1)^. Globally, the number of people with cataract-induced visual impairment is projected to approach 40 million by 2030 (https://nei.nih.gov/eyedata/cataract). Any type of opacity of the eye lens that affects normal vision are termed cataracts. The current mainstay treatment for cataracts is surgery, in the cloudy lens is extracted through phacoemulsification extracapsular cataract extraction (ECCE), followed by artificial intraocular lens (IOL) implantation. Overall, it is a cost-effective procedure for restoring the lost vision and has generally positive outcomes^(2)^.

Posterior capsule opacification (PCO) is a crucial complication of cataract surgery. PCO is also defined as a secondary cataract. PCO is caused by the proliferation and migration of lens epithelial cells (LECs) left on the anterior capsule and the capsular bag after cataract surgery^(3)^. These cells transdifferentiate into myofibroblast-­like cells during epithelial–mesenchymal transition (EMT). The factor that triggers the proliferation of residual LECs remains unclear, which makes targeting the EMT process therapeutically difficult. Hyaluronic acid (HA) is a naturally occurring macromolecular linear polysaccharide that interacts with surface receptors present in the extracellular matrix. HA can also be exogenously introduced during eye surgery through viscoelastic compounds, and this may affect the LECs and increase the rate of vitro PCO^(4)^. Increase in HA levels in epithelial cells may induce cytokeratin dispersion, loss of intercellular adhesion proteins, and vimentin upregulation, thereby resulting in EMT. Understanding the mechanism underlying potential HA-mediated EMT pathogenesis is crucial for preventing or treating this postsurgery complication.

The HA receptor CD44 has a putative role in mediating PCO pathogenesis^(4)^. It is expressed in numerous cell types and LECs of human eyes^(5)^, with known roles in modulating LEC proliferation in vitro^(6)^. HA binds to and activates CD44, thereby inducing signaling pathways that trigger cell proliferation and migration^(7)^. Among these pathways, transforming growth factor-β2 (TGF-β2) plays a crucial role in LEC modulation^(8)^. TGF-β has three isoforms regulating cellular functions, including cell growth^(9)^ and differentiation^(10)^. Several studies have indicated that CD44 promotes the FoxP3+ regulatory T cell persistence and function through TGF-β production^(11)^. In fibroblasts, CD44 regulates α-SMA gene expression through TGF-β signaling^(12)^ and inhibits cell proliferation^(6)^ by neutralizing CD44 antibodies. TGF-β induced EMT in lung cancer is linked to CD44 expression^(13)^. Additionally, CD44 silencing blocks TGF-β1-induced expression of stem cell-related factors in lung cancer^(13)^. CD44 interacts with the TGF signaling pathway and promotes the pro-fibrotic response of eye lens injury^(14)^. These findings suggest the potential interplay between TGF-β and CD44, which is relevant to cell proliferation and human disease.

Because TGF-β2 is the dominant isoform expressed in the human eye, we investigated whether HA and CD44 work through TGF-β2 in the pathogenic development of PCO. Using an in vitro model, we examined the role of TGF-β2 in the HA-mediated EMT of human LECs. Our results revealed that HLEB3 cells increased TGF-β2 production following HA treatment. Importantly, blocking CD44 expression prevented HA-mediated TGF-β2 upregulation. Our findings support a model where HA induces TGF-β2-dependent aberrant proliferation of LECs and identify CD44 as a potential therapeutic target in secondary cataract disease.

## METHODS

### Cell culture and transfection

Human LECs (HLEB3 cells) were purchased from ATCC (Rockville, MD., USA). The cells were cultured in Dulbecco’s modified Eagle medium (Gibco, Gaithersburg, MD, USA) supplemented with 10% fetal bovine serum (Gibco, USA) in a humidified 5% CO_2_ environment at 37°C. CD44-siRNA (Sangon Biotech, Shanghai, China) was used for CD44 silencing. According to the manufacturer’s instructions, the cells were seeded into 6-well plates for 24 h and infected with CD44-siRNA by using the Lipofectamine 3000 transfection reagent (Invitrogen, USA). The cells transfected for 24–72 h were then used for detection.

### Reagents and antibodies

HA was purchased from SINGCLEAN (Hangzhou, China), anti-CD44 was obtained from Proteintech (Wuhan, China), and anti-TGF-β2 was purchased from Abcam (Cambridge, MA, USA).

### Measurement of cell viability

The Cell Count Kit (CCK-8) (Beyotime, Shanghai, China) was used for measuring cell viability. HLEB3 cells (1 × 10^4^ cells well^−1^) were seeded overnight into 96-well plates. Then, the medium was exchanged for a serum-free medium containing increasing HA doses of 0.2, 0.4, 0.6, 0.8, 1.0, and 1.2 mg mL^−1^. After the plates were incubated for 24 h, 10 µL of WST-8 [2-(2-methoxy-4-nitrophenyl)-5-(2,4-disulfophenyl)-2H-tetrazolium monosodium salt] solution was added to each well. After the cells were incubated for 4 h at 37°C, cellular growth was examined at 450 nm by using a microplate reader (Molecular Devices, Sunnyvale, CA, USA). The number of living cells in each well was expressed as the measured value relative to the control.

### Scratch assay

HLEB3 cells (2 × 10^5^ cells well^−1^) were seeded into 6-well plates. On the second day, the cells were treated with HA. After 24 h of treatment, the cells in each well were scratched with a 200-µL pipette tip. The specific methods can be found in the literature^(15)^ 31259172. The images were captured using a digital camera (Olympus BX60, Tokyo, Japan). Ten fields of each plate were randomly marked. Gap width measurements were repeated in triplicate by using the same field.

### Immunohistochemistry

HLEB3 cells were cultured on chamber slides (Lab-Tek II, NY) and treated with HA for 24 h. Following the treatment, the cells were fixed with H_2_O_2_ in PBS at room temperature for 30 min. The fixed cells were permeabilized with 0.1% Triton X-100 for 15 min at room temperature, blocked with 5% normal goat serum for 1 h, and incubated with mouse-anti-TGF-β2 (1:100 dilution, Abcam) at 4°C for 48 h, followed by incubation with Alexa Fluor 488-goat antimouse IgG (1:250 dilution, Thermo Fisher, CN, USA) at 37°C for 1 h. The immunoreaction was measured by incubating the cells with horseradish peroxidase-labeled antibodies for 1 h at 37°C and visualized using the diaminobenzidine tetrachloride system (brown color). Images were captured using an Olympus BX60 microscope (Tokyo, Japan). The mean staining intensity of TGF-β2 was calculated using ImageJ software (NIH).

### Real-time quantitative polymerase chain reaction

Total RNA was isolated from the cells by using the Trizol reagent (Takara Bio Inc., Japan) and was reverse transcribed into cDNA according to the protocol of the PrimeScript RT Master Mix Perfect Real Time (Takara, Shiga, Japan). β-actin was used as an internal control for calculating the mRNA content. After the standard curve for quantitative polymerase chain reaction (qPCR) was constructed, the mRNA expression level was calculated. The results were generated using the 7300 Fast Real-Time PCR system (Applied Biosystems, Waltham, MA, USA).

The following primers were used for PCR:

β-actin, forward: TGTTACCAACTGGGACGACA,

reverse: CTTTTCACGGTTGGCCTTAG.

CD44, forward: GACACATAGCTCAATGCTTCAGC,

reverse: GATGCCAAGATGATCAGCCATTCTGCAAT.

TGF-β2, forward: GGAGCCTGAAGCAAGATTTGC,

reverse: TGCCAATGTAGTAGAGGATGGTGAG.

### Western blot analysis

Total cell lysates were prepared using the Mammalian Protein Extraction Reagent (Thermo Fisher, CN, USA). Extracted proteins (10-30 µg) were separated through sodium dodecyl sulfate-polyacrylamide gel electrophoresis and transferred onto a polyvinylidene fluoride membrane. The membranes were blocked with 5% blocking-grade non-fat milk in triethanolamine buffered saline solution and incubated with primary antibodies overnight at 4°C for 18 h, followed by incubation with secondary antibody (1:10,000; Odyssey) at room temperature for 1 h. Protein bands were detected using the Odyssey Infrared Imaging System (Li-Cor Biosciences) and quantified using ImageJ software.

### Statistical analysis

All experiments were performed at least in triplicates. Quantitative data are presented as the mean ± SEM after further analysis by conducting one-way ANOVA or Student’s t-test. P<0.05 was considered statistically significant.

## RESULTS

### Effects of HA on HLEB3 cell migration

**Figure 1:**
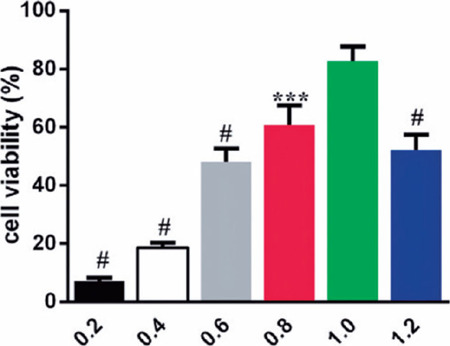
Optimum dose determination for HA treatment. HLEB3 cell viability was assessed using a commercial CCK8 kit after the cells were cultured for 24 h with increasing HA concentrations.

The viability of HLEB3 cells treated for 24 h with increasing HA concentrations was examined through the CCK-8 assay (Figure 1). The HLEB3 cell survival rate increased with increasing HA dosage. Cell viability peaked at 1.0 mg mL^−1^ HA and declined with 1.2 mg mL^−1^ HA (p<0.0001), suggesting cytotoxicity beyond this dose range. Thus, 1.0 mg mL^−1^ HA was used as the max effective dose to test for downstream effects in subsequent studies.

Data expressed as mean ± SEM. ***p<0.001, #p<0.0001 vs. concentration of 1.0 mg/mL.

**Figure 2:**
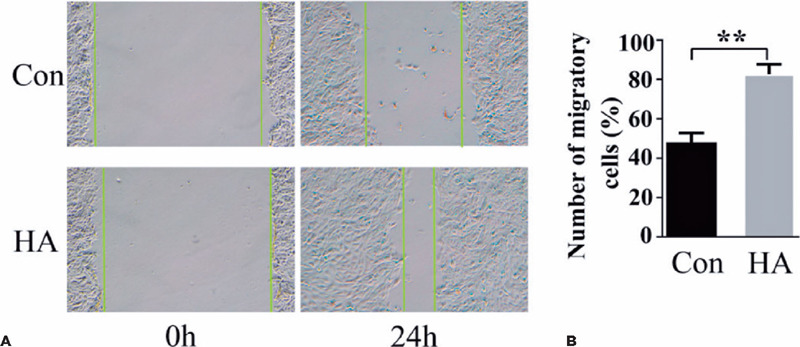
HA induced HLEB3 cell migration. (A) Time-lapse microscopy images of the scratch assay showing cells treated with control (Con) vs. HA at 0 and 24 h. Closure of the scratch indicated cell migration. (B) Quantitation of HLEB3 cell migration through the in vitro scratch assay. Cell migration was determined by counting the number of cells in the wound healing area.

HA is a component of the pericellular matrix found in many cell types that reorganizes cell migration and division^(16)^. Current studies have shown that exogenous HA can influence lens cell behavior^(17)^. To quantify the effects of HA on HLEB3 cell migration, a standard scratch test was conducted. In this test, we counted the number of cells that migrated into the scraped areas after 24 h (Figures 2A and 2B). Exogenous HA induced significantly greater cell migration in the treatment group than in the control group after 24 h in culture (p<0.01). These data suggest that exogenous HA promotes the mobility of human LECs in vitro.

Data expressed as mean ± SEM. **p<0.01 vs. the control group.

### CD44 silencing inhibits HA-induced HLEB3 cell proliferation and migration

**Figure 3:**
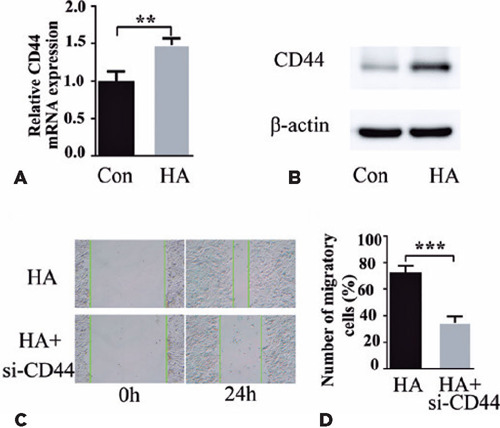
HA increased CD44 expression of HLEB3 cells. (A) The CD44 was mRNA level normalized to the β-actin mRNA level. (B) The CD44 protein level was measured through western blotting. (C, D) Microscopy images of the scratch assay and quantitative analysis.

The binding of HA to CD44 extracellular domains induces cell proliferation and migration^(18)^, thereby suggesting a mechanism through which HA triggers pathological cell proliferation in PCO. Cell treatment with HA for 24 h increased CD44 levels, as is evident from the qPCR data (Figure 3A), which exhibited an increase in CD44 mRNA levels. Correspondingly, CD44 protein induction was exhibited by western blotting (Figure 3B). Next, we evaluated the ability of CD44-siRNA to modulate HA-induced HLEB3 cell migration. In the cell scratch assay, CD44 knockdown strongly suppressed HA-induced HLEB3 cell migration (Figures 3C and 3D), which confirmed that HA acts through CD44 to modulate cell responses to wound healing. These findings suggest that HA induces the expression of its receptor CD44 in HLEB3 cells and that CD44 mediates the effect of HA on cell growth and migration.

Data expressed as mean ± SEM. **p<0.01, ***p<0.001 vs. the HA group.

### HA regulates TGF-β2 expression in HLEB3 cells through CD44

**Figure 4:**
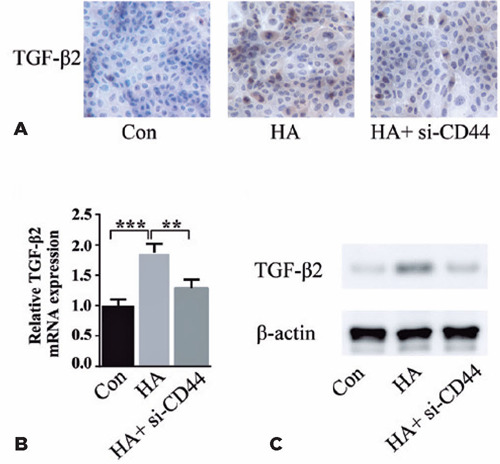
HA regulates TGF-β2 expression through CD44. (A) The TGF-β2 protein level in HLEB3 cells was detected through immunohistochemical staining (400×). (B) The TGF-β2 mRNA level was normalized to the β-actin mRNA level. (C) The TGF-β2 protein level as measured through western blotting.

To clarify the downstream mechanism underlying HA and CD44 action, we assessed whether this pathway is mediated through TGF-β2, an established effector of cell proliferation in PCO pathogenesis. Immunohistochemistry of HLEB3 cells revealed TGF-β2 induction by exogenous HA treatment, an effect mitigated by CD44 knockdown (Figure 4A). qPCR unveiled that HA increa­sed TGF-β2 mRNA levels and CD44 silencing could reverse the process (Figure 4B). HA treatment-mediated CD44 protein upregulation was reduced by blocking CD44 (Figure 4C). Results from these three lines of investigation suggested that HA regulates TGF-β2 expression in HLEB3 cells through CD44.

Data expressed as mean ± SEM. **p<0.01, ***p<0.001 vs. the HA group.

### The role of CD44 in TGF-β2 migration enhancement in LECs

**Figure 5:**
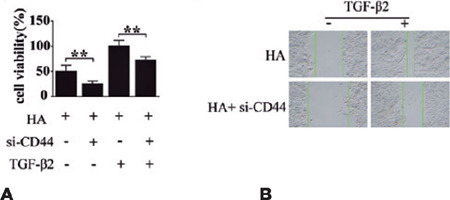
HA/CD44 regulates HLEB3 cell proliferation through TGF-β2. (A) The Cell Counting Kit (CCK-8) assay revealing that cell proliferation was the highest in cells treated with HA and/or TGF-β2, and that these effects were eliminated by CD44 knockdown. (B) Microscopy images of the scratch assay, showing CD44-dependent stimulation of cell migration by TGF-β2.

Many studies have indicated the role of active TGF-β2 in promoting the EMT of LECs^(19)^, and therefore, we investigated whether HA/CD44 participates in the regulation of the TGF-β2-mediated EMT of LECs. The CCK-8 and scratch assays revealed that CD44 silencing inhibited both HA-induced HLEB3 cell proliferation (Figure 5A) and migration (Figure 5B). These findings suggest that HLEB3 cell growth and migration depend on CD44, which in turn acts downstream of HA. The results revealed that CD44 might serve as a crucial mediator of the progression of TGF-β2-mediated EMT of LECs, in which HA is a key pathogenic factor.

Data expressed as mean ± SEM. **p<0.01.

## DISCUSSION

This study provides evidence consistent with an HA-CD44-TGF-β2 signaling axis in the pathological EMT of human cataracts. Although it may begin as part of normal healing, HA-induced cell proliferation and migration lead to aberrant changes in epithelial cells and cause postsurgery complications such as PCO. We here examined the molecular mechanism underpinning HA signaling in human LECs. Our results revealed that HA induces the EMT of LECs through the action of the HA receptor CD44. Moreover, TGF-β2 is a key signaling effector mediating the downstream effects of HA on cell proliferation and migration. Thus, HA works in concert with CD44 and TGF-β2 for promoting PCO formation through EMT regulation.

At present, surgery is the only effective method for eliminating cataracts and restoring vision in humans. However, the clinical utility of cataract surgery is limited by complications of secondary vision loss due to PCO formation after surgery. Proliferation, migration, and EMT of residual LECs causes PCO^(20)^. Chandler et al. demonstrated that introducing exogenous HA through surgical viscoelastic compounds possibly affects LEC and lens behavior, thus contributing to PCO^(4,17)^. Our data confirmed that exogenous HA induces HLEB3 cell proliferation and migration in a cell culture model. In various tissue types, HA modified cellular responses. This is the most notable capability of HA in EMT^(21)^. An exposure concentration of 1.0 mg mL^−1^ was used in our study to investigate the effect of surgical viscoelastic compounds during modern ECCE. Our results indicate that surgical viscoelastic compounds used at levels comparable to those actual used during cataract surgery may facilitate PCO formation through HA-mediated signaling mechanisms. Because of the potential sequelae of exogenous HA exposures, clinicians must use these compounds judiciously.

CD44, a cell surface receptor of HA, serves as an attractive target for pharmacological modulation. Regarding human eye diseases, CD44 is expressed in the aqueous humor^(22)^, the vitreous gel^(23)^, and the human lens^(24)^, with multiple intracellular functions in each cell type. CD44-neutralizing antibodies are effective at reducing LEC migration^(5)^, which suggests a functional role of CD44 in EMT and PCO formation, as well as the therapeutic potential of targeting this molecule in human diseases. We here observed that exogenous HA treatment upregulates CD44 expression. More importantly, silencing this induction by using CD44-siRNA reversed the detrimental signaling of HA and prevented HA treatment-induced cellular migration. These results suggest that CD44 is an endogenous mediator of HA-induced LEC migration and that targeting its expression or function is a viable approach for treating HA-related human eye diseases.

Our study links the HA/CD44 action to TGF-β2 signaling, a major regulatory pathway associated with cataract and PCO development^(25)^. TGF-β2 is involved in the EMT of LECs through both canonical^(26)^ and non-­canonical TGF pathways^(27)^. The functional significance of CD44/TGF-β2 signaling is underscored by the findings in other physiologic settings and tissue types, for example, in pulmonary fibroblasts, where CD44 is critical for migration-dependent TGF-β2 activation^(28)^, and in epicardial cells, where cell migration and invasion in response to TGF-β2 is at least partly mediated through CD44 signaling^(29)^. Literature on the regulatory role of CD44/TGF-β2 in tumor cell growth is growing^(30)^. CD44/TGF-β2 plays a prominent role in PCO formation, which may be relevant to human eye diseases, and thus offers a mechanistic understanding of HA action. These findings may facilitate in developing medical therapeutics to help reduce complications in patient after cataract surgery. Our study limitations include the use of human LEC lines in vitro rather than primary tissues, and the reliance on cell viability assays as an indirect measure of cell proliferation. The specific molecular mechanism through which TGF-β2 induces LEC migration also needs to be clarified. These questions and mechanisms remain to be addressed in future studies. Using multiple assay systems at both gene and protein levels, we showed that HA induces a process of CD44 and TGF-β2 activation, lea­ding to HLEB3 cell survival and increased mobility. Importantly, blocking of CD44 function allows the reversal of TGF-β2 induction and downstream effects of HA on PCO pathogenesis. In conclusion, our findings implicate CD44 and TGF-β2 as critical mediators of HA-­induced EMT in LECs. Future therapeutics targeting these molecules may aid in preventing HA-induced PCO.
